# Digital Inclusion and Women's Health Indicators Across Indian States: An Ecological Analysis of National Family Health Survey 2019-2021 Data

**DOI:** 10.7759/cureus.111289

**Published:** 2026-06-22

**Authors:** Hafsa Zahoor, Rameez Ahmed

**Affiliations:** 1 Obstetrics and Gynecology, Acharya Shri Chander College of Medical Sciences and Hospital, Jammu, IND; 2 Endocrinology and Metabolism, Sher-i-Kashmir Institute of Medical Sciences, Srinagar, IND

**Keywords:** anemia, cancer screening, contraception, digital inclusion, india, internet use, maternal health, menstrual hygiene, mobile phone, nfhs-5

## Abstract

Background: Digital inclusion may influence how women access health information and health services, but state-level evidence linking women's digital inclusion with women's health indicators in India remains limited. This study examined ecological associations between women's internet use, women's own mobile phone use, and selected women's health indicators across Indian states and union territories.

Methods: This ecological cross-sectional secondary analysis used an uploaded aggregated National Family Health Survey 2019-2021 (NFHS-5) state-level dataset. The India aggregate was excluded, leaving 36 states and union territories. Primary exposures were women who had ever used the internet and women having a mobile phone that they themselves use. Priority outcomes included menstrual hygiene, anemia, maternal health, contraception, and cancer screening indicators. Descriptive statistics, Pearson and Spearman correlations, simple linear regression, exploratory education-adjusted models, and NFHS-4 to NFHS-5 change analyses were conducted.

Results: Among 36 states and union territories, women's internet use ranged from 20.6% in Bihar to 76.7% in Sikkim, while women's own mobile phone use ranged from 38.5% in Madhya Pradesh to 91.2% in Goa. Menstrual hygiene was moderately and positively correlated with internet use (Pearson r = 0.615, p < 0.001) and mobile phone use (r = 0.583, p < 0.001). Anemia among women aged 15-49 years was negatively correlated with internet use (r = -0.432, p = 0.009) and mobile phone use (r = -0.464, p = 0.004). Maternal health, contraception, and cancer screening indicators showed weaker and less consistent associations. In exploratory models adjusted for women's schooling, the associations with menstrual hygiene and anemia were substantially attenuated and were no longer statistically significant.

Conclusion: Higher women's digital inclusion was ecologically associated with better menstrual hygiene and lower anemia prevalence across Indian states and union territories, but associations were weaker for several service-use outcomes. These findings are ecological and should not be interpreted as individual-level or causal evidence. Digital inclusion should be considered part of a broader social and health system context for women's health equity.

## Introduction

Digital technologies are increasingly embedded in health systems, including health information access, appointment navigation, telehealth, public health communication, and program monitoring. The World Health Organization has emphasized that digital health should support health and well-being while being guided by governance, infrastructure, financing, workforce readiness, and equity considerations [1]. Global connectivity data also show that digital access remains uneven, with persistent divides by sex, income, and geography [2]. Therefore, digital health equity depends not only on the availability of digital health interventions but also on whether women can access and use the internet, mobile phones, and related digital resources. Prior work on eHealth inequalities has warned that digital health tools may widen social health inequalities if access, literacy, affordability, and cultural barriers are not addressed [3,4].

India is an important setting for examining women's digital inclusion and health because of its large population, extensive state-level diversity, and continuing differences in education, connectivity, health service access, and women's autonomy. The National Family Health Survey 2019-2021 (NFHS-5) provides population, health, nutrition, reproductive health, women's empowerment, and biomarker indicators for India, states and union territories, and districts [5]. The wider Demographic and Health Surveys program has been used internationally as a comparable source of population and health data in low- and middle-income countries [6]. NFHS-5 includes indicators on women's internet use and women's own mobile phone use, making it possible to examine whether states with higher women's digital inclusion also have more favorable women's health profiles. An individual-level analysis of NFHS-5 reported substantial inequalities in internet use among women of reproductive age in India, with education, wealth, residence, and other social factors contributing to the distribution of internet access [7].

Women's digital inclusion may matter for health through several pathways, including access to health information, autonomy in communication, exposure to health promotion messages, and the ability to navigate health and social services. An exploratory NFHS analysis found that women's mobile phone access in India was associated with selected health service outcomes in urban settings, although phone access was unevenly distributed and did not uniformly improve rural health service use [8]. This suggests that digital access may be an enabling resource, but it is unlikely to operate independently of education, wealth, residence, service availability, and gender norms.

Several women's health domains measured in NFHS-5 are particularly relevant to digital inclusion and public health equity. Menstrual health is increasingly understood as a broader state of physical, mental, and social well-being related to the menstrual cycle, rather than only the use of products [9]. Evidence from India shows that menstrual hygiene practices are associated with education, household resources, media exposure, residence, and state or district context [10-12]. Anemia among women remains a major public health concern in low- and middle-income countries and is shaped by nutritional, infectious, reproductive, and socioeconomic factors [13,14]. India has implemented the Anaemia Mukt Bharat strategy to strengthen prevention and control through iron-folic acid supplementation, testing and treatment, behavior change communication, supply chain measures, and monitoring [15].

Cancer screening, maternal health, and family planning are also relevant because they depend on information, awareness, service access, provider recommendation, and health system capacity. NFHS-5-based analysis has reported very low cancer screening uptake in India [16], while national and global guidance emphasize organized screening pathways for common cancers, including cervical cancer [17,18]. Evidence from NFHS-5 also indicates that adequate quality antenatal care remains unevenly distributed [19] and that unmet need for family planning declined from NFHS-4 to NFHS-5 but remained geographically and socially patterned [20].

Despite these individual-level and program-specific studies, less is known about how women's digital inclusion and women's health indicators are distributed together at the state and union territory level. Consequently, this study had two objectives: (1) to evaluate ecological associations between women's digital inclusion, measured through women's internet use and women's own mobile phone use, and selected domains of women's health, including menstrual hygiene, anemia, maternal healthcare, contraception, and cancer screening, and (2) to examine whether associations for the principal outcomes were attenuated after exploratory adjustment for women's schooling. Menstrual hygiene and anemia were treated as the principal outcomes, while maternal health, contraception, and cancer screening indicators were examined as secondary exploratory domains. Because this was an exploratory secondary analysis and was not preregistered, no directional hypothesis was specified. The findings were interpreted exclusively at the state or union territory level.

## Materials and methods

Study design and reporting approach

This study was designed as an ecological, cross-sectional secondary analysis of aggregated state-level NFHS-5 data. The unit of analysis was the Indian state or union territory, not the individual respondent. The study was reported using relevant principles from the Strengthening the Reporting of Observational Studies in Epidemiology statement, particularly transparent reporting of study design, data source, variables, statistical methods, and limitations [21]. Because the study used aggregate data, ecological associations were interpreted at the state or union territory level only. Ecological studies compare groups rather than individuals and cannot estimate the individual-level joint distribution of exposures and outcomes [22].

Data source and analytic sample

The analysis used the NFHS-5-States csv file [23], a secondary aggregation derived from official NFHS-5 state factsheets. The file contained aggregated NFHS-5 and selected NFHS-4 indicators, including state, state code, indicator name, NFHS-5 urban, rural, and total values, and NFHS-4 total values. The official NFHS-5 report and factsheets published by the International Institute for Population Sciences (IIPS) were treated as the authoritative sources for the survey scope and indicator definitions [5].

The India aggregate row was excluded to avoid analyzing the national estimate together with its component states and union territories. The final analytic sample included 36 states and union territories.

The NFHS-5 was conducted during 2019-2021 and provides representative estimates for India, states and union territories, and districts across domains including population, reproductive health, maternal and child health, nutrition, women's empowerment, and biomarker indicators [5]. For each selected indicator, the NFHS-5 total value was used as the primary analytic value. Urban and rural values were used for descriptive availability checks. NFHS-4 total values were used only where comparable values were available in the secondary dataset.

Variables

The primary exposure variables were women who had ever used the internet (%) and women having a mobile phone that they themselves use (%). These indicators were selected because they directly measured women's digital inclusion in the secondary dataset. Women with 10 or more years of schooling (%) were used as a contextual covariate in exploratory adjusted models because education is strongly related to digital access and women's health outcomes.

Outcomes were selected after inspecting all indicator labels in the dataset. Menstrual hygiene and anemia were treated as the principal outcome domains because they represented the main health and health-related behavioral indicators examined in relation to both digital inclusion exposures. Menstrual hygiene was measured using the proportion of women aged 15-24 years who used hygienic methods of protection during their menstrual period. Anemia indicators included anemia among all women aged 15-49 years, pregnant women aged 15-49 years, and women aged 15-19 years.

Maternal healthcare, contraception, and cancer screening indicators were treated as secondary exploratory domains. Maternal health indicators included first-trimester antenatal care, at least four antenatal care visits, maternal postnatal care within two days, institutional births, and births attended by skilled health personnel. Contraception indicators included the use of any modern contraceptive method and total unmet need for family planning. Cancer screening indicators included cervical cancer screening and breast examination for breast cancer. This hierarchy distinguished the principal ecological patterns from the broader set of hypothesis-generating analyses.

Blood pressure, blood sugar, and duplicated oral cancer screening indicators were not included because the secondary dataset contained duplicated or insufficient sex-specific labels for these indicators. These indicators would require verification against the original NFHS tables before use as women-specific outcomes.

Data management and statistical analysis

The source comma-separated value (CSV) was retained unchanged, and a separate cleaned analytic dataset was created. Indicators were selected by exact matching to their numbered NFHS factsheet labels. The India aggregate was removed, percentage fields were converted to numeric format, and the data were reshaped from long to wide format, with one row per state or union territory and one column per selected indicator. Duplicate indicator labels were reviewed manually. Blood pressure, blood sugar, and duplicated oral cancer screening indicators were excluded because the aggregated file did not contain sufficiently clear sex-specific labels. Blank values were treated as missing, no values were imputed, and analyses used available observations for each indicator pair or regression model.

Descriptive statistics were calculated for each selected indicator, including the mean, standard deviation (SD), minimum, maximum, and the corresponding states or union territories. Ecological associations between the digital inclusion exposures and selected outcomes were assessed using Pearson and Spearman correlation coefficients. Pearson correlation was used to assess linear relationships between state-level percentages, while Spearman correlation provided a rank-based assessment of monotonic relationships.

Simple ordinary least squares regression models estimated the unadjusted association between each digital inclusion exposure and each selected health outcome. Regression coefficients were interpreted as the expected percentage-point difference in the outcome associated with a one percentage-point higher value of the digital inclusion indicator at the state or union territory level.

Exploratory adjusted models were fitted for the principal menstrual hygiene and anemia outcomes by adding women with 10 or more years of schooling (%) as a contextual covariate. These models examined whether the principal unadjusted associations were attenuated after accounting for state-level differences in women's schooling. The adjusted models were interpreted cautiously because the ecological sample was small and the digital inclusion indicators were strongly correlated with women's schooling. They were not interpreted as estimating independent causal effects.

NFHS-4 to NFHS-5 change values were calculated as the NFHS-5 total value minus the NFHS-4 total value for indicators with values available in both survey rounds. Positive values indicated an increase between the two surveys, while negative values indicated a decrease.

All statistical tests were two-sided, and p-values below 0.05 were considered statistically significant. Because multiple ecological associations were examined, p-values were interpreted alongside effect sizes, consistency between Pearson and Spearman estimates, and substantive plausibility. No adjustment for multiple comparisons was applied.

All analyses reported in this manuscript were conducted using Python version 3.13.5. Data management and descriptive analyses were performed using pandas version 2.2.3 and NumPy version 2.3.5. Pearson and Spearman correlation analyses were conducted using SciPy version 1.17.0. Ordinary least squares regression models, including coefficients, 95% confidence intervals (CIs), p-values, and R-squared values, were estimated using statsmodels version 0.14.6. Figures were generated using Matplotlib version 3.10.8.

## Results

This ecological analysis identified substantial geographic co-variation between women's digital inclusion and selected women's health indicators across India. In unadjusted analyses, states and union territories with higher women's internet use and mobile phone use tended to report higher menstrual hygiene and lower anemia prevalence. However, these associations were substantially attenuated and were no longer statistically significant after exploratory adjustment for women's schooling. This attenuation indicates that the digital inclusion indicators may largely reflect broader state-level socioeconomic development rather than an independent digital association. Maternal healthcare, contraception, and cancer screening indicators showed weaker and less consistent ecological relationships.

The analytic dataset included 36 states and union territories after excluding the India aggregate. NFHS-5 total values were available for all 36 analytical units for the two digital inclusion exposures and for most priority outcomes. Pregnant women's anemia had one missing state or union territory value, while cancer screening, menstrual hygiene, most contraception indicators, and most maternal service indicators had complete NFHS-5 total values.

Women's digital inclusion varied substantially across states and union territories. The mean proportion of women who had ever used the internet was 43.35% (n = 36, SD: 16.35), ranging from 20.6% in Bihar to 76.7% in Sikkim. Women's own mobile phone use had a mean of 65.09% (n = 36, SD: 15.19), ranging from 38.5% in Madhya Pradesh to 91.2% in Goa. Descriptive statistics for selected indicators are shown in Table 1.

**Table 1:** Descriptive statistics for digital inclusion and selected women's health indicators across Indian states and union territories Values are NFHS-5 total percentages calculated across 36 states and union territories after excluding the India aggregate. SD: standard deviation, NFHS: National Family Health Survey

Indicator	Number	Mean (%)	SD	Lowest value	Highest value
Women who have ever used the internet	36	43.35	16.35	20.6 (Bihar)	76.7 (Sikkim)
Women having a mobile phone they themselves use	36	65.09	15.19	38.5 (Madhya Pradesh)	91.2 (Goa)
Women with 10 or more years of schooling	36	46.50	13.34	23.2 (Tripura)	77.0 (Kerala)
Women aged 15-24 using hygienic menstrual protection	36	83.78	11.87	58.8 (Bihar)	99.1 (Puducherry)
All women aged 15-49 with anemia	36	53.99	13.57	25.8 (Lakshadweep)	92.8 (Ladakh)
Pregnant women aged 15-49 with anemia	35	47.98	12.34	20.9 (Lakshadweep)	78.1 (Ladakh)
Women aged 15-19 with anemia	36	55.75	14.03	27.9 (Manipur)	96.9 (Ladakh)
First-trimester antenatal checkup	36	73.49	11.37	49.5 (Nagaland)	99.6 (Lakshadweep)
At least four antenatal care visits	36	65.86	17.99	20.7 (Nagaland)	93.0 (Goa)
Maternal postnatal care within two days	36	79.73	13.70	43.9 (Meghalaya)	95.4 (Goa)
Institutional births	36	89.37	11.58	45.7 (Nagaland)	99.8 (Kerala)
Births attended by skilled health personnel	36	90.56	9.54	55.3 (Nagaland)	100.0 (Kerala)
Any modern contraceptive method	36	52.99	12.35	18.2 (Manipur)	70.8 (Andhra Pradesh)
Total unmet need for family planning	36	10.10	4.05	4.7 (Andhra Pradesh)	26.9 (Meghalaya)
Cervical cancer screening	36	1.73	2.22	0.2 (Assam)	9.8 (Tamil Nadu)
Breast examination for breast cancer	36	0.80	1.20	0.0 (Chandigarh)	5.6 (Tamil Nadu)

Ecological correlations between digital inclusion and priority outcomes are presented in Table 2. Menstrual hygiene showed moderate positive correlations with both women's internet use (Pearson r = 0.615, p < 0.001; Spearman's rho = 0.617, p < 0.001; n = 36) and women's own mobile phone use (Pearson r = 0.583, p < 0.001; Spearman's rho = 0.567, p < 0.001; n = 36). Anemia among all women aged 15-49 years showed moderate negative correlations with internet use (Pearson r = -0.432, p = 0.009; n = 36) and mobile phone use (Pearson r = -0.464, p = 0.004; n = 36). Pregnant women's anemia also showed negative associations with both digital inclusion indicators (n = 35).

**Table 2:** Ecological correlations between women's digital inclusion and priority women's health indicators Correlations are unadjusted and based on NFHS-5 total percentages. P-values were not adjusted for multiple comparisons. NFHS: National Family Health Survey

Outcome	Number	Pearson r internet	p	Spearman's rho internet	p	Pearson r mobile	p	Spearman's rho mobile	p
Women aged 15-24 using hygienic menstrual protection	36	0.615	<0.001	0.617	<0.001	0.583	<0.001	0.567	<0.001
All women aged 15-49 with anemia	36	-0.432	0.009	-0.555	<0.001	-0.464	0.004	-0.553	<0.001
Pregnant women aged 15-49 with anemia	35	-0.536	<0.001	-0.683	<0.001	-0.559	<0.001	-0.659	<0.001
Women aged 15-19 with anemia	36	-0.395	0.017	-0.544	<0.001	-0.450	0.006	-0.570	<0.001
At least four antenatal care visits	36	0.292	0.084	0.334	0.046	0.328	0.051	0.401	0.015
Institutional births	36	0.183	0.285	0.315	0.062	0.058	0.739	0.338	0.043
Any modern contraceptive method	36	-0.124	0.471	-0.112	0.516	-0.245	0.149	-0.215	0.209
Total unmet need for family planning	36	0.078	0.649	0.138	0.424	0.225	0.186	0.278	0.101
Cervical cancer screening	36	0.223	0.191	0.239	0.161	0.293	0.082	0.295	0.081
Breast examination for breast cancer	36	0.263	0.121	0.139	0.417	0.389	0.019	0.362	0.030

**Figure 1:**
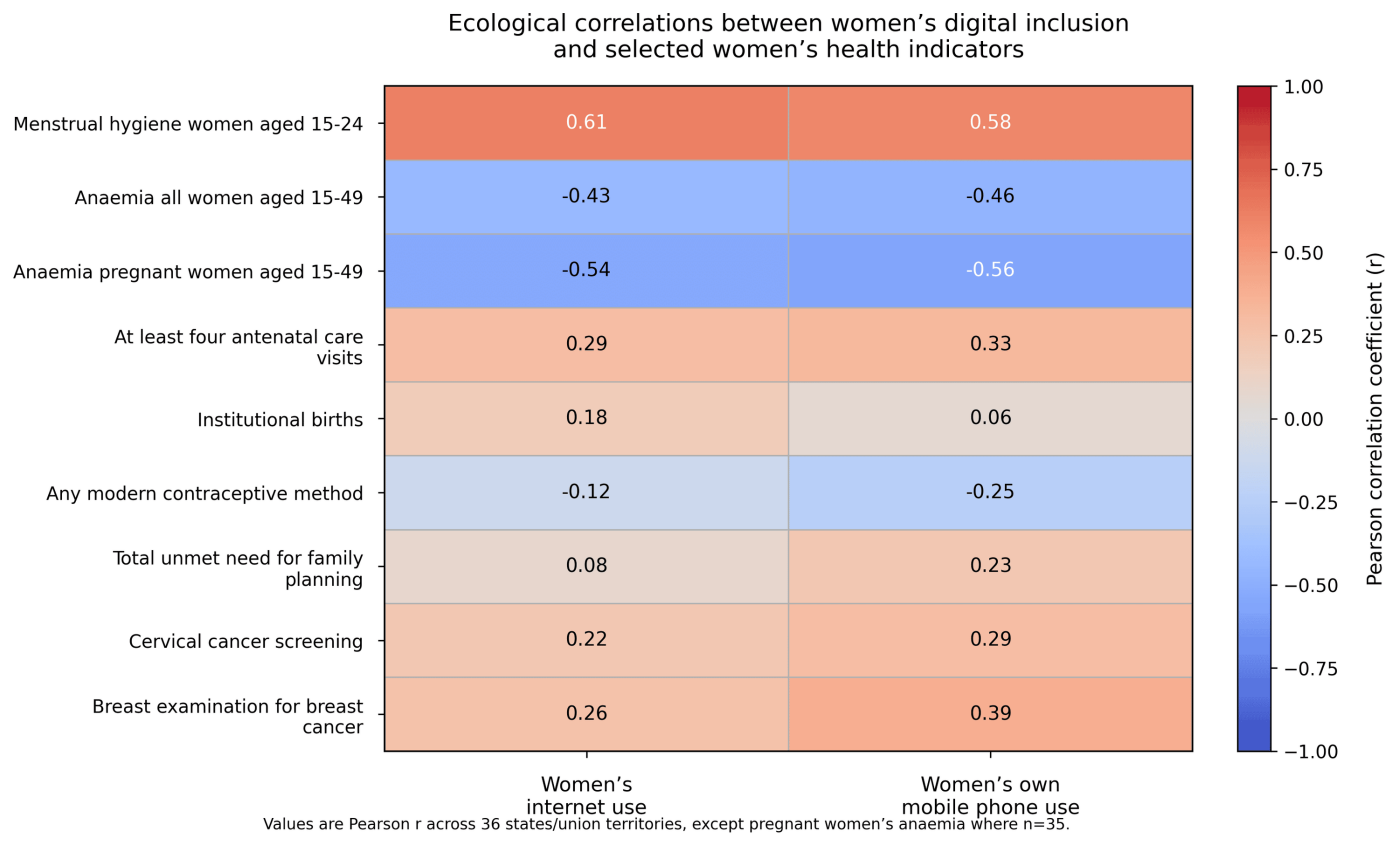
Heatmap of Pearson correlations between women's digital inclusion and selected outcomes Cells show unadjusted Pearson correlation coefficients between each digital inclusion exposure and selected women's health indicators. Positive values indicate that higher digital inclusion was associated with higher outcome values; negative values indicate that higher digital inclusion was associated with lower outcome values.

The state-level scatterplots in Figure 1 visualize the strongest digital inclusion associations observed in the correlation results. 

**Figure 2:**
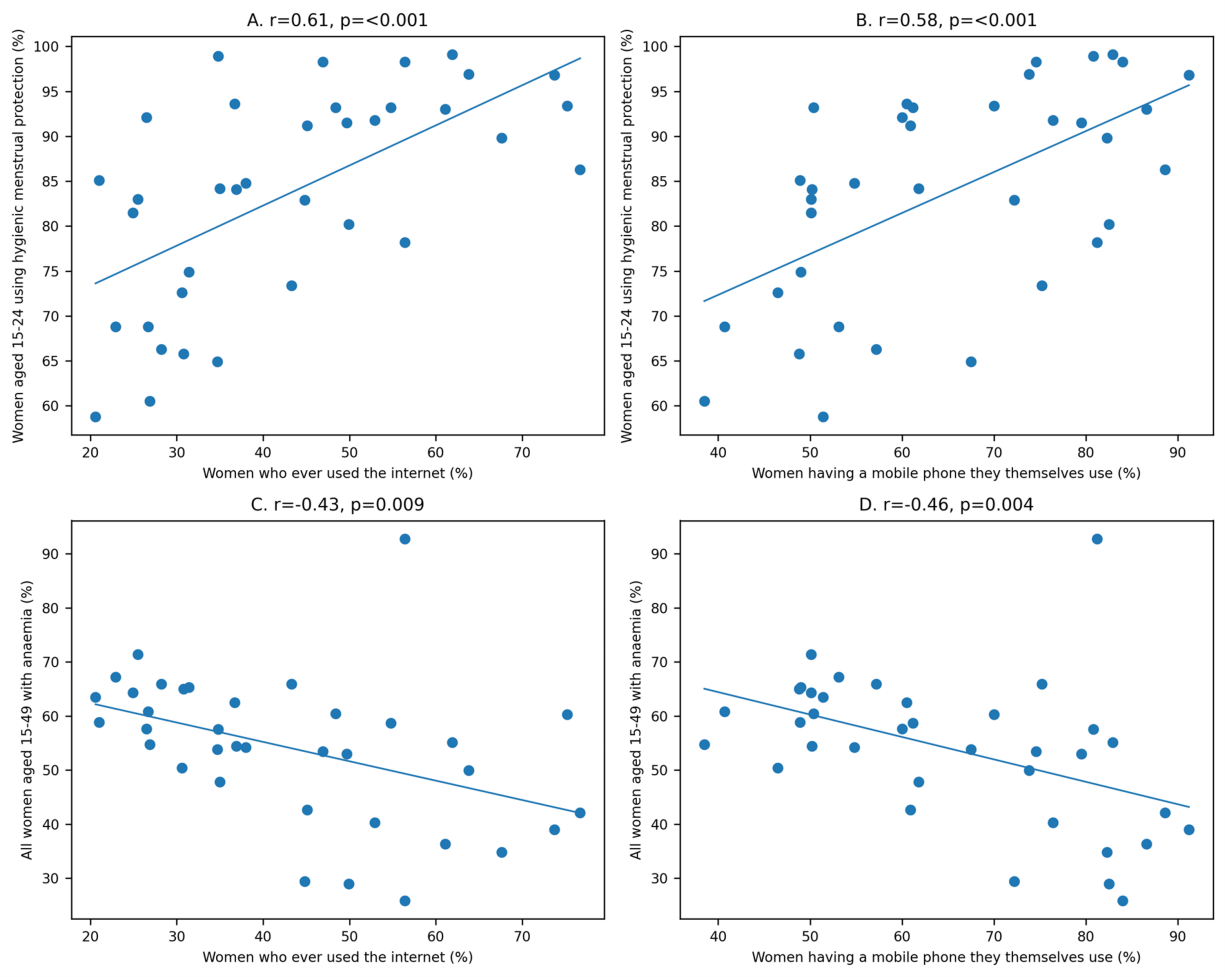
State-level associations between women's digital inclusion, menstrual hygiene, and anemia Panel A shows women's internet use and menstrual hygiene; panel B shows women's own mobile phone use and menstrual hygiene; panel C shows women's internet use and anemia among all women aged 15-49 years; panel D shows women's own mobile phone use and anemia among all women aged 15-49 years. Each point represents one state or union territory.

The Pearson correlation heatmap in Figure 2 summarizes the broader pattern across digital inclusion exposures and selected outcomes.

Most maternal health service indicators showed weaker or non-significant Pearson correlations with digital inclusion. At least four antenatal care visits showed weak positive Pearson correlations with internet use (r = 0.292, p = 0.084; n = 36) and mobile phone use (r = 0.328, p = 0.051; n = 36). Spearman correlations for at least four antenatal care visits were slightly stronger (internet rho = 0.334, p = 0.046; n = 36; mobile rho = 0.401, p = 0.015; n = 36). Institutional births and skilled birth attendance showed weak or very weak Pearson correlations with digital inclusion, although rank-based correlations were positive for institutional births with mobile phone use (rho = 0.338, p = 0.043; n = 36) and skilled birth attendance with both internet use (rho = 0.392, p = 0.018; n = 36) and mobile phone use (rho = 0.375, p = 0.024; n = 36). For contraception, modern method use showed weak negative Pearson correlations, while total unmet need showed weak positive Pearson correlations. Cancer screening uptake was low and showed weak associations overall; breast examination had a weak positive correlation with mobile phone use (r = 0.389, p = 0.019; n = 36), while cervical cancer screening had weak correlations with both exposures. The corresponding simple ecological regression models are summarized in Table 3.

**Table 3:** Unadjusted ecological linear regression models for selected outcomes Models are unadjusted. Beta represents the percentage-point difference in the outcome associated with a one percentage-point higher exposure value at the state or union territory level. CI: confidence interval

Outcome	Exposure	Number	Beta	95% CI	p	R-squared
Women aged 15-24 using hygienic menstrual protection	Women who ever used the internet	36	0.446	0.247 to 0.646	<0.001	0.378
Women aged 15-24 using hygienic menstrual protection	Women having a mobile phone they themselves use	36	0.456	0.235 to 0.677	<0.001	0.340
All women aged 15-49 with anemia	Women who ever used the internet	36	-0.359	-0.620 to -0.098	0.009	0.186
All women aged 15-49 with anemia	Women having a mobile phone they themselves use	36	-0.415	-0.691 to -0.139	0.004	0.216
Pregnant women aged 15-49 with anemia	Women who ever used the internet	35	-0.423	-0.659 to -0.187	<0.001	0.287
Pregnant women aged 15-49 with anemia	Women having a mobile phone they themselves use	35	-0.448	-0.684 to -0.213	<0.001	0.312
At least four antenatal care visits	Women who ever used the internet	36	0.321	-0.045 to 0.688	0.084	0.085
At least four antenatal care visits	Women having a mobile phone they themselves use	36	0.388	-0.002 to 0.778	0.051	0.107
Institutional births	Women who ever used the internet	36	0.130	-0.113 to 0.372	0.285	0.034
Institutional births	Women having a mobile phone they themselves use	36	0.044	-0.221 to 0.309	0.739	0.003
Any modern contraceptive method	Women who ever used the internet	36	-0.094	-0.355 to 0.167	0.471	0.015
Any modern contraceptive method	Women having a mobile phone they themselves use	36	-0.200	-0.474 to 0.075	0.149	0.060
Total unmet need for family planning	Women who ever used the internet	36	0.019	-0.067 to 0.106	0.649	0.006
Total unmet need for family planning	Women having a mobile phone they themselves use	36	0.060	-0.030 to 0.151	0.186	0.051
Cervical cancer screening	Women who ever used the internet	36	0.030	-0.016 to 0.077	0.191	0.050
Cervical cancer screening	Women having a mobile phone they themselves use	36	0.043	-0.006 to 0.092	0.082	0.086
Breast examination for breast cancer	Women who ever used the internet	36	0.019	-0.005 to 0.044	0.121	0.069
Breast examination for breast cancer	Women having a mobile phone they themselves use	36	0.031	0.005 to 0.056	0.019	0.151

In simple regression models (Table 3), each one percentage-point higher level of women's internet use was associated with a 0.446 percentage-point higher menstrual hygiene value (95% CI: 0.247 to 0.646, p < 0.001; n = 36). Each one percentage-point higher level of women's own mobile phone use was associated with a 0.456 percentage-point higher menstrual hygiene value (95% CI: 0.235 to 0.677, p < 0.001; n = 36). For anemia among all women aged 15-49 years, each one percentage-point higher level of internet use was associated with a 0.359 percentage-point lower anemia value (95% CI: -0.620 to -0.098, p = 0.009; n = 36), while each one percentage-point higher level of mobile phone use was associated with a 0.415 percentage-point lower anemia value (95% CI: -0.691 to -0.139, p = 0.004; n = 36).

In exploratory models adjusted for women with 10 or more years of schooling, the associations between digital inclusion and the principal outcomes were substantially attenuated. For menstrual hygiene, the adjusted coefficient was 0.076 for women's internet use (95% CI: -0.188 to 0.341, p = 0.561; n = 36) and 0.044 for women's own mobile phone use (95% CI: -0.233 to 0.321, p = 0.749; n = 36). For anemia among all women aged 15-49 years, the adjusted coefficient was -0.190 for internet use (95% CI: -0.595 to 0.214, p = 0.346; n = 36) and -0.274 for mobile phone use (95% CI: -0.691 to 0.143, p = 0.190; n = 36). Women's internet use and women's own mobile phone use were strongly correlated with women having 10 or more years of schooling at the state level (Pearson r = 0.765 and r = 0.748, respectively). These exploratory findings indicate that the unadjusted associations may substantially reflect broader educational and socioeconomic differences across states and union territories rather than an independent digital association.

NFHS-4 to NFHS-5 change values for indicators with paired data are summarized in Table 4.

**Table 4:** Change from NFHS-4 to NFHS-5 for selected indicators with available values Change was calculated as NFHS-5 total minus NFHS-4 total. Positive values indicate an increase from NFHS-4 to NFHS-5. NFHS: National Family Health Survey

Indicator	Number	Mean change (percentage points)	Lowest change	Highest change
Women having a mobile phone they themselves use	36	9.65	-4.2 (Chandigarh)	21.3 (Jammu and Kashmir)
Women aged 15-24 using hygienic menstrual protection	36	14.06	-3.6 (Mizoram)	34.1 (Odisha)
At least four antenatal care visits	36	4.11	-16.3 (Sikkim)	30.9 (Uttarakhand)
Institutional births	36	8.18	-0.3 (Puducherry)	27.0 (Arunachal Pradesh)
Any modern contraceptive method	36	8.20	-16.7 (Ladakh)	35.3 (Goa)
Total unmet need for family planning	36	-4.26	-17.9 (Manipur)	5.7 (Meghalaya)
All women aged 15-49 with anemia	36	2.72	-20.2 (Lakshadweep)	19.9 (Assam)

Where NFHS-4 values were available, women's own mobile phone use increased by a mean of 9.65 percentage points, and menstrual hygiene increased by a mean of 14.06 percentage points. Changes in maternal health service indicators and contraception indicators were generally smaller. Anemia among all women aged 15-49 years increased by a mean of 2.72 percentage points across states and union territories with available values.

## Discussion

Principal findings

This ecological analysis identified substantial geographic variation and co-variation between women's digital inclusion and selected women's health indicators across India. In unadjusted analyses, states and union territories with higher women's internet use and mobile phone use tended to report greater use of hygienic menstrual protection and lower anemia prevalence. However, these associations were substantially attenuated and were no longer statistically significant after exploratory adjustment for women's schooling, suggesting that they may largely reflect broader state-level educational and socioeconomic development rather than an independent digital association. Maternal healthcare, contraception, and cancer screening indicators showed weaker and less consistent relationships with digital inclusion.

The strongest unadjusted positive association was between digital inclusion and menstrual hygiene. This relationship is plausible because menstrual hygiene practices are influenced by access to information, education, affordability, social norms, product access, and exposure to media or health promotion messages. Prior studies in India have reported that hygienic menstrual practices are patterned by education, wealth, place of residence, mass media exposure, and geography [10-12]. Our study adds a state-level digital inclusion perspective, showing that states with higher women's internet and mobile phone use also tended to have higher menstrual hygiene values. However, the attenuation observed after adjustment for women's schooling indicates that this relationship may substantially reflect broader educational and socioeconomic conditions. The findings do not show that individual women used digital technologies to obtain menstrual health information or products.

The negative ecological association between digital inclusion and anemia should also be interpreted cautiously. Anemia is multifactorial and influenced by diet, iron deficiency, infection, inflammation, reproductive factors, sanitation, poverty, and health system performance [13,14]. Digital inclusion is unlikely to directly reduce anemia by itself. More plausibly, digital inclusion may act as a marker of broader social advantage, including education, household resources, urbanization, infrastructure, and health system performance. This interpretation is supported by the strong state-level correlations between the digital inclusion indicators and women's schooling, as well as the attenuation of the anemia associations in the schooling-adjusted models.

Maternal health service indicators showed weaker associations with digital inclusion. This suggests that digital inclusion does not translate uniformly across all women's health domains at the state level. Antenatal care, institutional delivery, skilled birth attendance, and postnatal care depend heavily on service availability, facility quality, transport, community health workers, program delivery, and local health system capacity. Recent NFHS-5-based research on adequate quality antenatal care similarly highlights the importance of service content and socioeconomic factors, rather than attendance alone [19]. Digital access may therefore be relevant to information and service navigation, but is insufficient to explain geographic variation in maternal healthcare indicators.

Cancer screening indicators also showed weak associations with digital inclusion. This is not unexpected because screening uptake in India remains very low, and organized screening pathways require trained providers, accessible facilities, referral systems, diagnostic confirmation, and follow-up [16,17]. Digital tools may support awareness, reminders, navigation, and follow-up, but general access to the internet or a mobile phone is unlikely to overcome structural barriers to screening. This is particularly important for cervical cancer, for which global elimination targets depend on high screening coverage and effective treatment of precancerous lesions [18].

Family planning indicators showed weak and statistically non-significant associations with digital inclusion. This should not be interpreted as evidence that targeted mHealth family planning interventions are ineffective. The exposures examined in this study measured general state-level internet use and personal mobile phone use, rather than participation in an intervention designed to influence contraceptive knowledge, initiation, or continuation. Reviews of mHealth interventions for contraception and sexual and reproductive health have reported mixed findings and emphasized the relevance of tailored content, interactive communication, privacy, implementation quality, and linkage to appropriate services [24,25]. At the state level, modern contraceptive use and unmet need may be more strongly influenced by fertility preferences, parity, method availability, commodity supply, counselling quality, partner and household dynamics, and regional program history. Although digital access may facilitate information and communication, it cannot by itself ensure that preferred contraceptive methods, confidential counselling, or accessible services are available. Aggregation across entire states may also conceal associations within particular age, marital, socioeconomic, or geographic groups.

Implications for digital health equity

These findings have implications for digital health equity. Digital health interventions may offer additional channels for health education, reminders, counselling, service navigation, and monitoring, but they can also exclude women with limited connectivity, lower digital literacy, inadequate privacy, less autonomy, or fewer economic resources [3,4]. In India, women with internet and mobile phone access may be concentrated in states and population groups with stronger educational and socioeconomic conditions [7,8]. Digital inclusion should therefore be understood as part of a wider enabling environment rather than as an isolated determinant of women's health.

Menstrual hygiene and anemia may be promising domains for integrated digital and public health strategies, but digital interventions should remain embedded within broader programs. For menstrual hygiene, digital channels could support age-appropriate education, stigma reduction, information about hygienic products, and linkage to school or community-based services. For anemia, digital tools could support reminders, counselling, supply monitoring, and follow-up, but should complement nutrition, supplementation, testing, treatment, infection control, and social protection measures [15].

The weaker associations for cancer screening, maternal healthcare, and family planning indicate that digital inclusion should not be treated as a substitute for health system strengthening. Public health departments should adopt a digital-plus rather than digital-only approach, consistent with guidance that digital interventions should complement functioning health services and be assessed for equity, feasibility, and acceptability [26]. Mobile campaigns should provide equivalent low-technology and non-digital pathways, such as basic text messaging, toll-free voice or interactive voice services, printed local-language information, and counselling through community health workers and primary healthcare services. Registration and access should not depend on personal smartphone ownership. Assisted access could be provided through health facilities, schools, women's groups, and community outreach.

Content should also be designed for low bandwidth, local languages, varying literacy levels, offline use, and shared-device environments. Privacy protections are particularly important for menstrual, reproductive, and cancer-related communications when women share phones with partners or family members. Public health departments should monitor campaign reach, referral completion, and follow-up across rurality, age, education, socioeconomic position, and device ownership, and strengthen community-based or non-digital outreach where digitally excluded groups are under-reached.

Strengths and limitations

This study has several strengths. It used a nationally recognized public data source and included all Indian states and union territories as ecological units. It focused on women-specific digital inclusion indicators and a broad set of women's health domains, including menstrual hygiene, anemia, maternal healthcare, contraception, and cancer screening. The analysis was deliberately conservative, using descriptive statistics, correlations, and simple regression rather than complex modeling that would be inappropriate for a small ecological sample. Exploratory adjustment for women's schooling also provided additional insight into the likely influence of broader social development on the observed relationships.

The study also has important limitations. First, the ecological design means that associations apply to states and union territories, not to individual women. The findings cannot show that an individual woman's internet or mobile phone use improves menstrual hygiene, reduces anemia, or increases health service use. Relationships observed between aggregate indicators may differ from relationships at the individual level.

Second, the analytic sample included only 36 units, limiting statistical power and the number of covariates that could be included reliably. Women's internet use and personal mobile phone use may function as markers of wider state-level development, capturing education, household wealth, urbanization, infrastructure, women's autonomy, and health system capacity. The substantial attenuation of the principal associations after adjustment for women's schooling supports this interpretation. The source dataset did not contain a directly comparable state-level wealth or economic development measure, and residual confounding by wealth and other contextual factors could not be addressed. The analysis, therefore, cannot establish that digital inclusion has an association with women's health that is independent of broader socioeconomic development.

Third, internet use and mobile phone use are basic indicators of digital inclusion. They do not capture digital literacy, smartphone ownership, frequency or purpose of internet use, connectivity quality, affordability, privacy, online safety, or actual exposure to digital health interventions. In particular, general digital access should not be treated as equivalent to participation in a targeted mHealth program.

Fourth, the selected outcomes had different denominators. Some indicators related to all women aged 15-49 years, some to women aged 15-24 years, some to pregnant women, and others to births or recent mothers. These indicators are appropriate for domain-specific ecological analysis but should not be interpreted as measuring the same underlying population.

Finally, the analysis relied on a third-party aggregation derived from official NFHS-5 state factsheets rather than official IIPS microdata. The repository documentation indicates that the factsheets were converted into a structured dataset with limited quality checking and recommends verification against the original source. Although the calculations were internally checked against the analyzed CSV and official IIPS sources were used for survey scope and indicator definitions, every state-by-indicator value was not independently re-entered from the official factsheets. Data extraction, transcription, or conversion errors may therefore remain possible.

## Conclusions

Across Indian states and union territories, women's internet use and personal mobile phone use co-varied with menstrual hygiene and anemia in unadjusted analyses, but these relationships were substantially attenuated after adjustment for women's schooling. Digital inclusion indicators are therefore likely to capture broader differences in education, wealth, urbanization, infrastructure, and health system performance. No independent digital or causal effect can be established from this ecological study. Associations with maternal healthcare, contraception, and cancer screening were weak or inconsistent. Digital health programs should use digital-plus delivery models that combine low-bandwidth and assisted digital options with community-based and non-digital pathways. Future individual-level analyses should assess whether digital access remains associated with women's health after adjustment for education, household wealth, residence, caste, age, autonomy, and service availability.
